# Buffalo Medical and Surgical Association

**Published:** 1886-04

**Authors:** 


### Buffalo Medical and Surgical Association.

*Meeting of March 3, 1886.*

As the President and Vice-President were absent, Dr. M. Hartwig was elected chairman *pro tem.* Present—Drs. Brecht,

Grosvenor, Burkhardt, Chas. Ring, Coakley, Davis, Dorland, Eli Long, Van Peyma, Wm. G. Ring, Campbell, Fowler, and, by invitation, Drs. Steele and Matzinger.

Dr. Grosvenor was proposed for membership.

Dr. Wm. G. Ring, the essayist of the evening, read a paper upon "*Renal Hemorrhage*," describing a case which had occurred in his practice, and giving method of diagnosis and treatment.

DISCUSSION.

Dr. Coakley mentioned the fact that hematuria was confined to certain localities. He had observed it more frequently in Buffalo than in Virginia, where he formerly practiced, and attributed it to the large amount of lime salts in Niagara water. He claimed that boiling the water would in a measure remove these salts.

Dr. Hartwig noticed that the essayist had made no mention of a microscopic examination, which he considered very important in making a diagnosis. If the hematuria was of renal origin, the blood corpuscles would appear very indistinctly, or not at all.

Dr. Potter recalled a case of renal hemorrhage due to traumatism, a wagon heavily loaded having passed over a man. The hemorrhage lasted two weeks, when the patient recovered.

*Voluntary Communications.*—Dr. Hartwig reported the case of a boy, six years of age, who suffered from a total suppression of urine. There had previously been diphtheria; there was no dropsy. Collapse, resembling that seen in cholera, occurred, followed by death. Post mortem examination showed that death was due to acute nephritis. Microscopic specimens were presented, showing various forms of casts.

*Prevailing Diseases.*—Pneumonia, bronchitis, and influenza.

